# Clinical-radiomic models based on digital breast tomosynthesis images: a preliminary investigation of a predictive tool for cancer diagnosis

**DOI:** 10.3389/fonc.2023.1152158

**Published:** 2023-05-12

**Authors:** Federica Murtas, Valeria Landoni, Pedro Ordòñez, Laura Greco, Francesca Romana Ferranti, Andrea Russo, Letizia Perracchio, Antonello Vidiri

**Affiliations:** ^1^ Medical Physics Department, IRCCS Regina Elena National Cancer Institute, Rome, Italy; ^2^ Department of Biomedicine and Prevention, University of Rome "Tor Vergata", Rome, Italy; ^3^ Radiology and Diagnostic Imaging Department, IRCCS Regina Elena National Cancer Institute, Rome, Italy; ^4^ Pathology Department, IRCCS Regina Elena National Cancer Institute, Rome, Italy

**Keywords:** radiomic, predictive model, breast cancer, AI, tomosynthesis (DBT)

## Abstract

**Objective:**

This study aimed to develop a clinical–radiomic model based on radiomic features extracted from digital breast tomosynthesis (DBT) images and clinical factors that may help to discriminate between benign and malignant breast lesions.

**Materials and methods:**

A total of 150 patients were included in this study. DBT images acquired in the setting of a screening protocol were used. Lesions were delineated by two expert radiologists. Malignity was always confirmed by histopathological data. The data were randomly divided into training and validation set with an 80:20 ratio. A total of 58 radiomic features were extracted from each lesion using the LIFEx Software. Three different key methods of feature selection were implemented in Python: (1) K best (KB), (2) sequential (S), and (3) Random Forrest (RF). A model was therefore produced for each subset of seven variables using a machine-learning algorithm, which exploits the RF classification based on the Gini index.

**Results:**

All three clinical–radiomic models show significant differences (p < 0.05) between malignant and benign tumors. The area under the curve (AUC) values of the models obtained with three different feature selection methods were 0.72 [0.64,0.80], 0.72 [0.64,0.80] and 0.74 [0.66,0.82] for KB, SFS, and RF, respectively.

**Conclusion:**

The clinical–radiomic models developed by using radiomic features from DBT images showed a good discriminating power and hence may help radiologists in breast cancer tumor diagnoses already at the first screening.

## 1 Introduction

Breast cancer (BC) is the tumor with the highest incidence worldwide. With 2.3 million new cases estimated in 2020, it represents the 1.7% of new cancer diagnoses and is therefore the most frequently diagnosed according to Global Cancer Statistics 2020 (1). Although screening and advancements in personalized treatments have led to an improvement in survival rates, it is estimated that BC-related deaths will increase 43% globally from 2015 to 2030 (2).

In current radiological practice, mammographic, ultrasonographic, or magnetic resonance imaging (MRI) evaluation of tumors is largely qualitative and includes subjective evaluations such as tumor aspect (spiculated, rounded, with necrosis, microcalcification), density, type of enhancement and anatomic relationship to the surrounding tissues in order to inform further treatment (3, 4). Early studies have shown that the three-dimensional (3D) digital mammography (DBT) examination can lead to an effective reduction in both false-positive and false-negative rates when compared with traditional X-ray mammography for all breast density subtypes (5, 6), especially on heterogeneously dense breasts.

The inclusion of standard digital imaging among the possible sources of big data for precision medicine represents one of the new frontiers of research. Particularly, radiomics (7) offers a great opportunity for diagnosis in several medical fields, yielding the most interesting results in oncology. Radiomics aims to extract quantitative information, which is potentially beyond the perception of the human eye, from medical images to uncover novel features that are associated with treatment outcomes, disease molecular expressions, and/or patient survival (8).

There are few studies concerning the analysis of DBT images and eventually the introduction into clinical practice of methods of automatic cancer detection (9–12). However, the scientific interest in radiomics and artificial intelligence (AI) methods in this setting is rapidly expanding. In this scenario, the aim of our project was the construction of a predictive model of lesion malignancy based on the radiomic features extracted by DBT images and on the clinical and anatomopathological characteristics of the lesions, which could assist radiologists in their first level diagnosis.

## 2 Material and methods

In the present study, patients who were subjected to tomosynthesis exams were enrolled; DBT imaging was performed at the Breast Unit in the Department of Radiology and Diagnostic Imaging by using the Giotto^®^ CLASS mammography unit. Images were transferred from the picture archiving communication system (PACS) to a dedicated MIM-Maestro system (MIM Software INC.) in which the lesion was identified by the radiologists. This study was approved by the IRCCS Regina Elena Cancer Institute Ethics Committee (CEI number: RS1414/20(2408)). The requirement for obtaining informed consent was waived as it was a retrospective study.

### 2.1 Patient inclusion

In this study, 150 patients who underwent DBT scans were enrolled, 80 of whom had lesions classified as malignant and 70 benign. Lesions radiologically classified as malignant were subsequently confirmed by pathologic analysis.

Patients were randomly collected among those undergoing DBT at our hospital from May 2021 to May 2022, and their characteristics were quite extensively distributed as shown in **Table 1**. Those who had previously undergone radio/chemo or immunotherapy treatment or breast surgery and whose regions of interest (ROIs) couldn’t be segmented due to artifacts in the DBT image were excluded.

**Table 1:** Clinical characteristics.

Age (mean ± SD)	Benign (70) 54.04 ± 13.14	Malignant (80)66.36 ± 15.21
Density:		
**A**	15	27
**B**	26	33
**C**	25	17
**D**	4	3
Bi-Rads:		
**1-2**	38	0
**3**	26	12
**4a-4b-4c**	6	50
**5**	0	18

### 2.2 Tomosynthesis acquisition/visualization protocol

Parameters for performing DBT scans were selected automatically by the automatic exposure control (AEC) at fixed Target/filter combination (W/Ag 50 ± 5 µm). The images resolution was 2925 × 1342 pixels per each 1 mm reconstructed slice. The initial images reading was performed on a workstation with diagnostic quality monitors (BARCO 5 MP).

### 2.3 Dataset allocation

The data were randomly divided into the training and validation sets in a ratio of 80:20. In the subdivision process, attention was paid to maintaining the predetermined relationship between patients of one group or the other.

### 2.4 Lesion contouring

Lesions were always identified by two expert radiologists (more than 10 years of experience). In the case of a very irregular shape, often a malignant lesion, the radiologist manually performed the contouring. Otherwise, a semi-automatic contouring method was applied. In both cases, the most representative DBT slice was chosen according to the radiologist’s indication.

**Figure 1:**
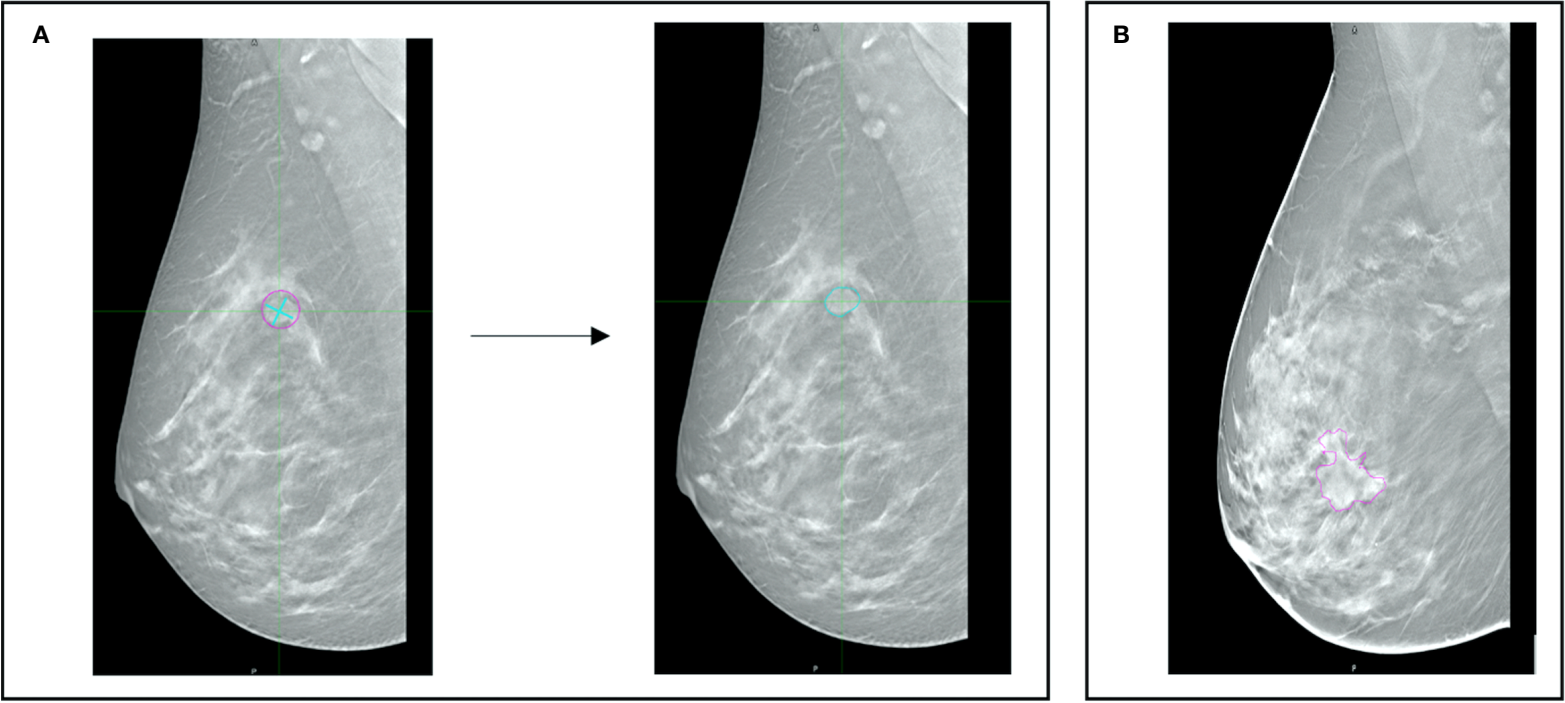
**(A)** Example of the automatic contouring method: the operator identifies the center of the lesion and draws a cross to signal to the software the size of the area of interest. The algorithm then draws a contour based on the density gradients obtained from the values of the pixels of interest. **(B)** manually delineated lesion.

An example of delineated lesions is shown in **Figure 1**.

The algorithm, called “2D Edge”, is part of the MIM-Maestro system (MIM Software INC.). The density gradient method was used to draw a particular region of the image previously identified by the operator. To assess the robustness of the algorithm, a specific lesion of 2.84 cm^2^ manually delineated by an expert radiologist was also automatically contoured 20 times, resulting in a median area value of 2.83 cm^2^ (range 2.69–2.99). Furthermore, a qualitative validation regarding the shape was performed.

### 2.5 Collection of clinical variables

Patient clinical data, such as age, breast density, and Breast Imaging–Reporting and Data System (BI-RADS) scores were collected in a dedicated database specifically created with Microsoft Access software.

The lesion-associated anatomical and pathological data were obtained from the characterization following the biopsy, and data, such as estrogen, progesterone, human epidermal growth factor raptor 2 (HER2) and Ki67 (13) based on the histological evaluation, were also included in the same database.

Malignant or benign status of the lesions was defined according to a breast screening report [NHSBSP https://www.gov.uk/government/publications/breast-screening-national-radiographic-workforce-report-2016]:

- B1 not adequate or not representative/probable the lesion was not taken.
- B2 benign.
- B3 with atypia but probably benign.
- B4 with suspected atypia but not diagnostic for malignancy.
- B5 malignant (B5a carcinoma *in situ*, B5b infiltrating carcinoma, B5c all malignant non-epithelial neoplasms).

### 2.6 Collection of radiomic variables

The images and the contours of the lesion exported from MIM were transferred to an open access software that allowed the extraction of radiomic features: LIFEx (www.lifexsoft.org). The analysis took place using first and second order radiomic features.

A total of 58 features were extracted from the original images: (1) five in the shape category, (2) 22 first-order statistical features and (3) 31 textural (n=6 Gray Level Co-occurrence Matrix [GLCM] + 11 Gray Level Run Length Matrix [GLRM] + 3 Neighboring Gray Level Dependence Matrix [NGLDM] + 11 Gray Level Size Zone Matrix [GLZLM]). All extracted features were obtained from the original image without any kind of filter. LIFEx output was an Excel file containing for each row all the variables extracted from one lesion analysis.

### 2.7 Development of a clinical-radiomic model

The clinical–radiomic model was constructed by combining age and density with the 58 cited features associated with each lesion. The ratio between training and validation set was chosen trying to balance the two groups (10) while maintaining a reasonable number of patients to train the model (14). The model was then developed with the training set (80% of the patients, n = 120) and tested using the validation set (20% of the patients, n = 30) splitted using a five-fold cross validation method.

#### 2.7.1 Features selection

The number of features suitable for representing the population was chosen considering the dimension and the variability of the sample. Usually, it is considered a good practice to take a number of features in the ratio 1:9 respect to the sample size, to avoid possible overfitting seven features were selected from the initial 60 to build the model. Three different key methods of feature selection have been implemented in Python and included K best (KB), sequential (S), and Random Forest (RF).

The KB is based on a filter method (15). In filter methods, features are selected independently from any machine algorithms using a specific criterion, such as scores in statistical test and variances, to rank the importance of individual features. These methods are also generally effective in computation time and that’s why they are mainly used as the pre-processing step of any feature selection pipeline.

To estimate the degree of linearity between the input features (such as predictor of malignancy) and the output feature, the analysis of variance (ANOVA) F-value method was implemented. To avoid issues with outliers and violations of distributional assumptions, all features were previously normalized using a normal transformation of the ranks. However, any non-linear relationships cannot be detected by ANOVA F-value. Hence in the S method, to avoid and capture also non-linear relationships between input and output features a Mutual information (MI) algorithm was implemented (16). The S method is a wrapper method that finds the best subset of feature by adding a feature at each iteration that best improves the accuracy of the model. The maximum number of features must be set as an input.

The main weakness of filter methods is the lack of consideration of the relationships among features. To obtain a robust model but at the same time not overburden it, it is necessary to discard the information that turns out to be overwhelming. In fact, if two characteristics are strongly correlated, it is sufficient to consider only one for the construction of the final model. This information can be derived by creating a correlation matrix between the characteristics.

The last feature selection method used is an Embedded method, RF (15). It combines the strong points of filter and wrapper methods by taking advantage of machine algorithms that have their own built-in feature selection process.

#### 2.7.2 Machine learning algorithm

Three models were therefore produced, one for each subset of variables, through a machine-learning algorithm, implemented in python language, which exploits the Random Forrest classification based on the Gini index (17). Models M1–3 were obtained from three feature subsets selected by KB, S, and RF, respectively.

### 2.8 Statistical analysis

The Mann–Whitney (18) test was used to assess the differences between selected features, both clinical and radiomic. All significance tests were considered under α=5% (p-value ≤ 0.05).

The goodness of the three models obtained was compared by analyzing the area under the curve (AUC) of the receiver operating characteristic (ROC) curve (19).

**Figure 2:**
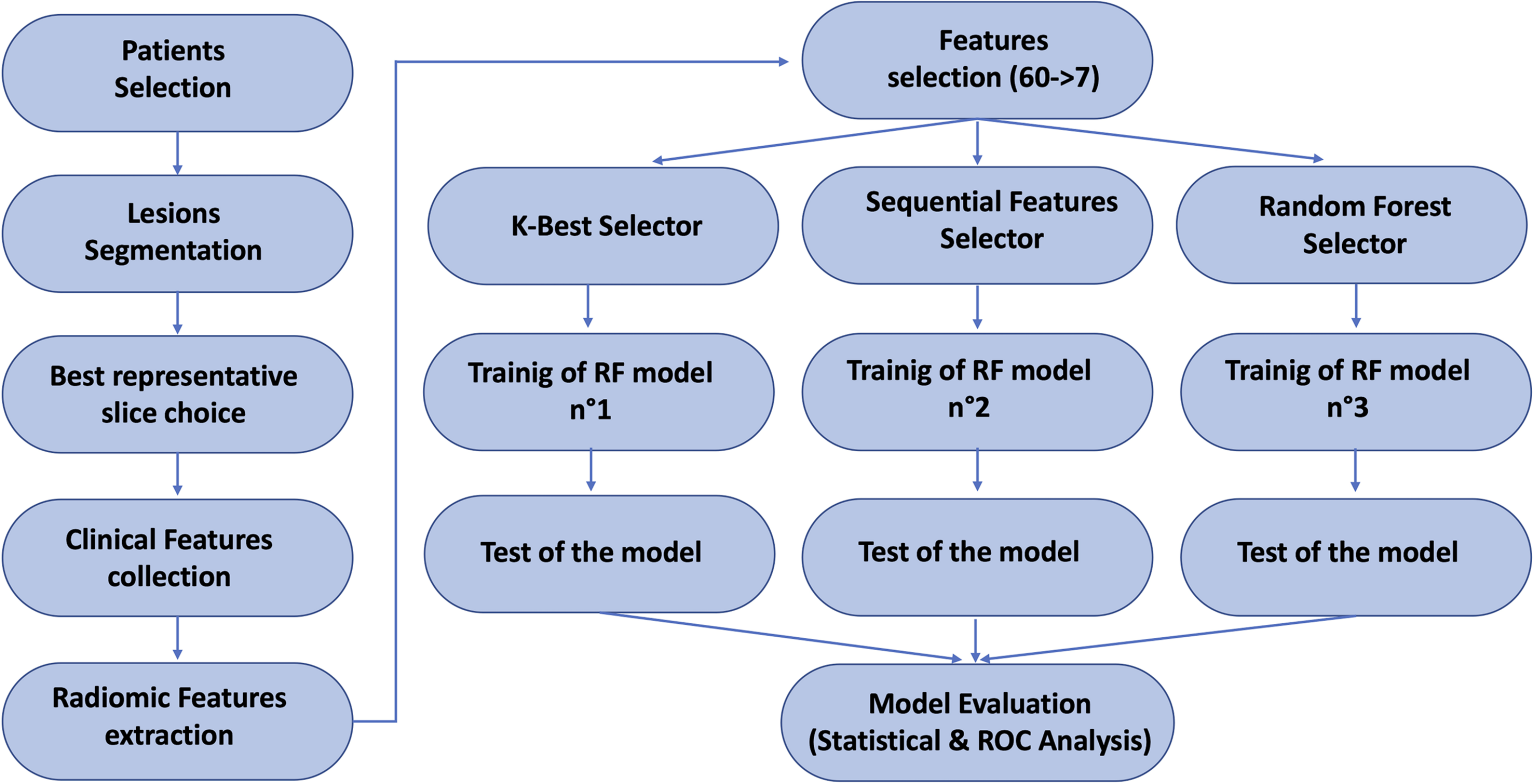
Scheme of the overall pipeline of this study.

**Figure 2** illustrates the workflow of this study.

## 3 Results

In **Table 1**, the clinical characteristics of the enrolled patients are reported.

**Figure 3:**
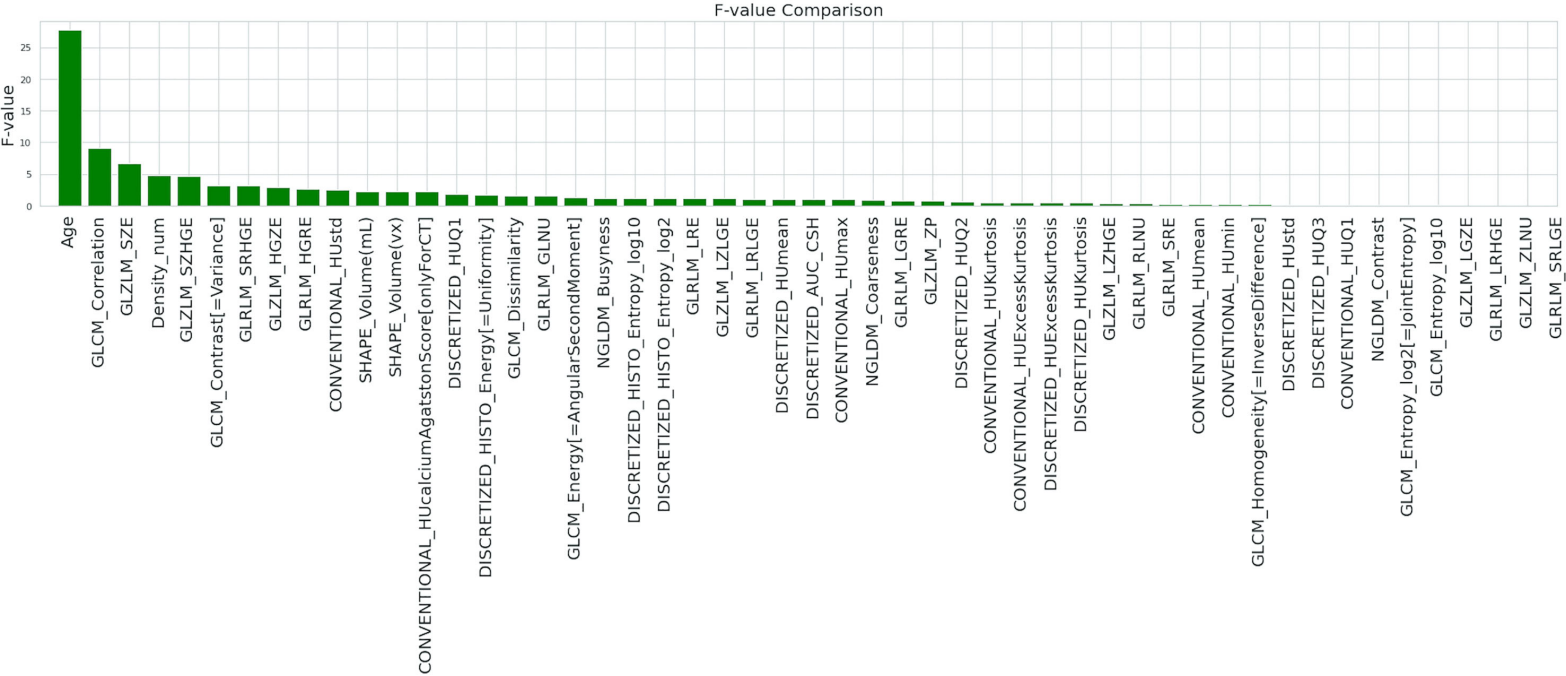
Result of the F-test on the input variables when using the KB filter method.

In **Figure 3**, results of the analysis of variance (ANOVA) F-value method applied when using the K-best (KB) filter method are shown.

A high F-value indicates high degree of linearity, and a low F-value indicates a low degree. The presence of some promising variables (such as age and GLCM_Correlation) and others not correlated with the dichotomous output variable was immediately visible.

**Figure 4:**
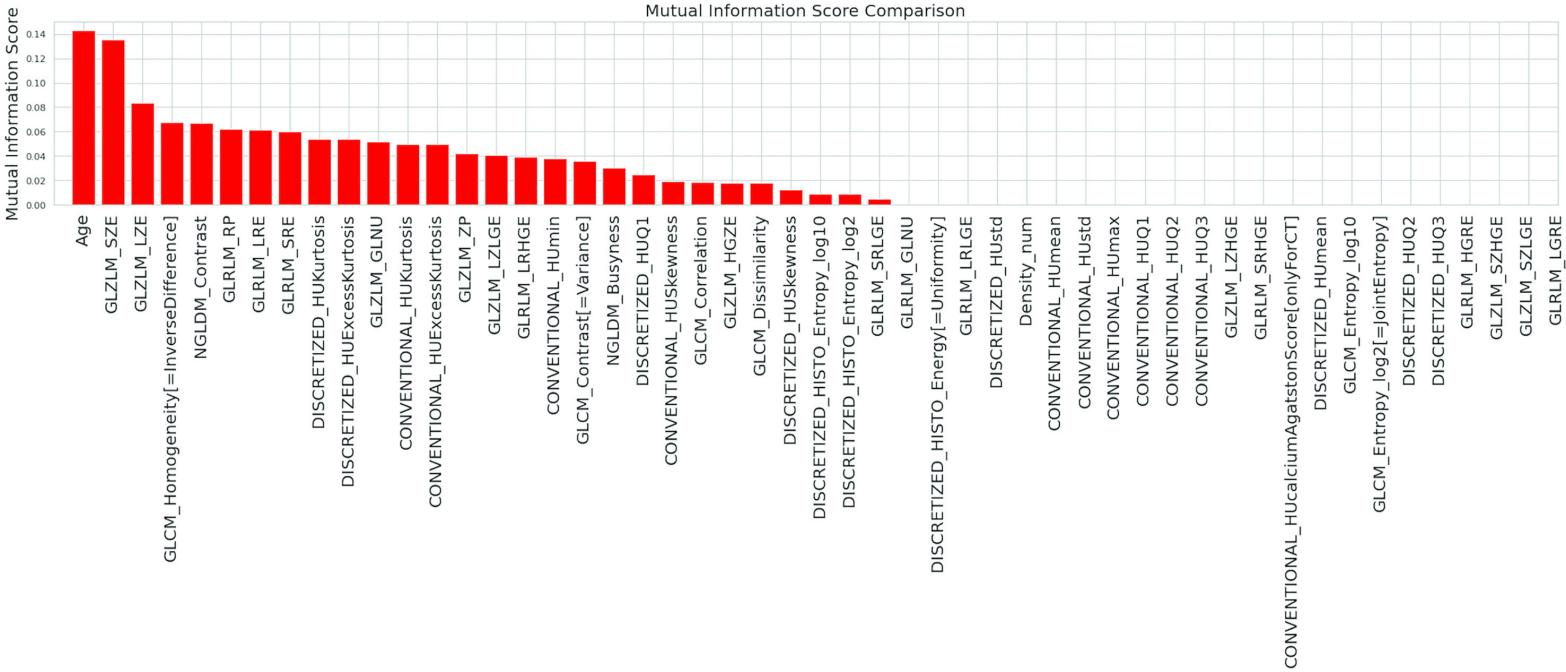
Mutual Information scores between output and input variables when using the S filter method.

In the S filter method, MI measures the dependence, also non-linear, of one variable to another by quantifying the amount of information obtained about one feature through the other. MI is symmetric and non-negative; it is zero only if the input and output features are independent (**Figure 4**).

**Figure 5:**
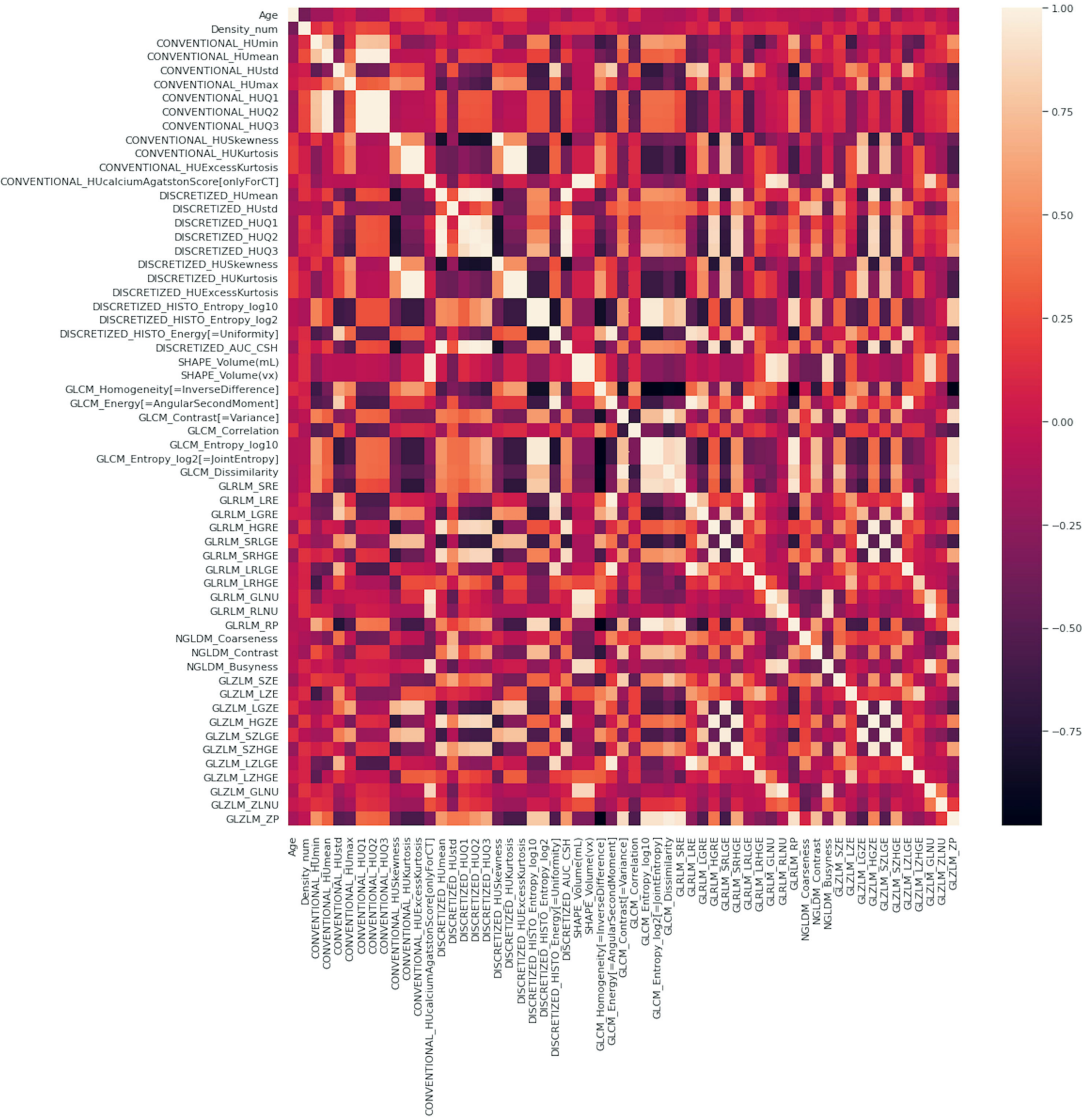
Correlation matrix of the complete set of variables, clinical plus radiomic.

A correlation matrix, such as the one shown in **Figure 5** in which the inter-variable dependence was highlighted through the chromatic scale, was created to eliminate highly colinear variables in the feature selection step for the creation of the final model.

**Figure 6:**
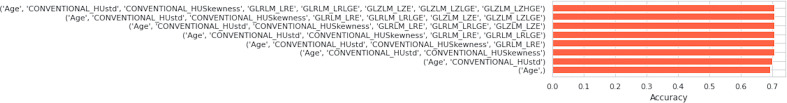
Accuracy plot showed with respect of the subset of features considered.

In **Figure 6** the accuracy calculated by the S method to choose the most predictive variables is shown. Best accuracy of 0.72 is reached with all the seven variables.

Finally, the RF method was used.

**Figure 7:**
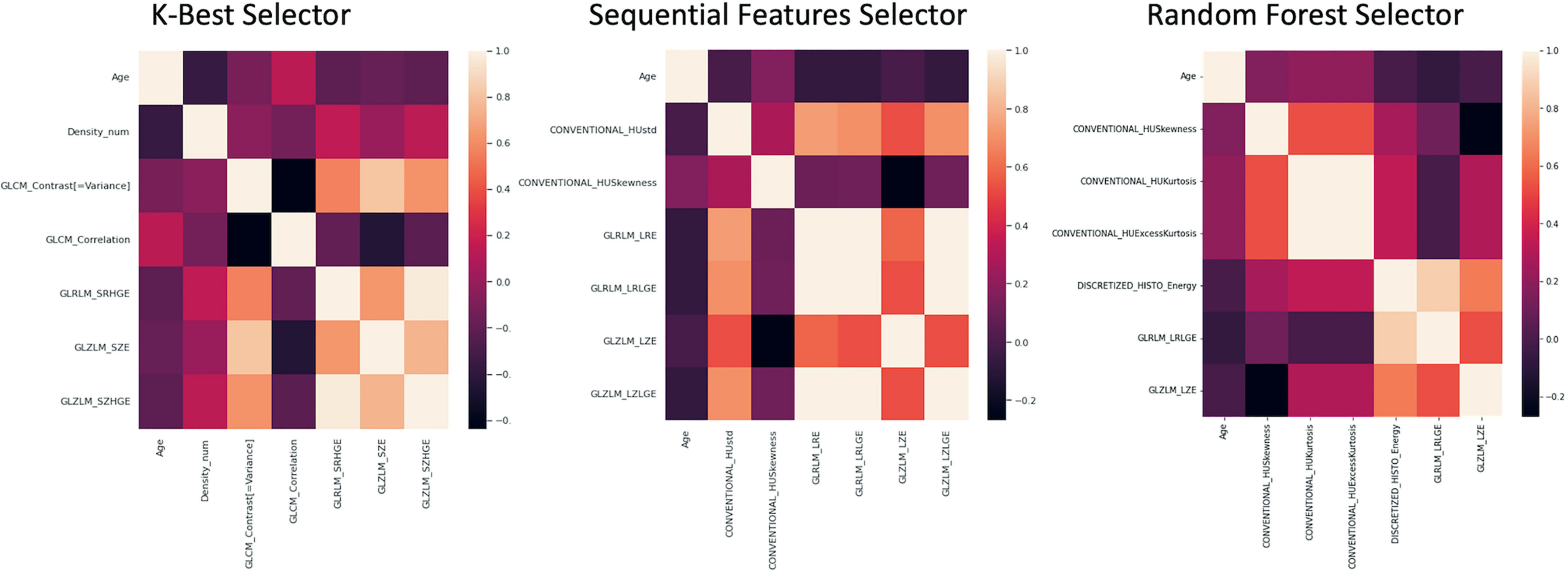
Correlation matrices of the 7 features obtained by KB, S and RF selection method are shown.

In **Figure 7** the seven features obtained by KB, S, and RF selection methods are shown.

Based on the Mann-Whitney test, the following features resulted to be significantly different (p < 0.05) between benign and malignant lesions: (1) age, (2) density, (3) CONVENTIONAL_HUKurtosis, (4) CONVENTIONAL_HUExcessKurtosis, (5) GLCM correlation, (6) GLRLM_LRLGE, (7) GLRLM_SZE, and (8) GLRLM_SZHGE.

**Figure 8:**
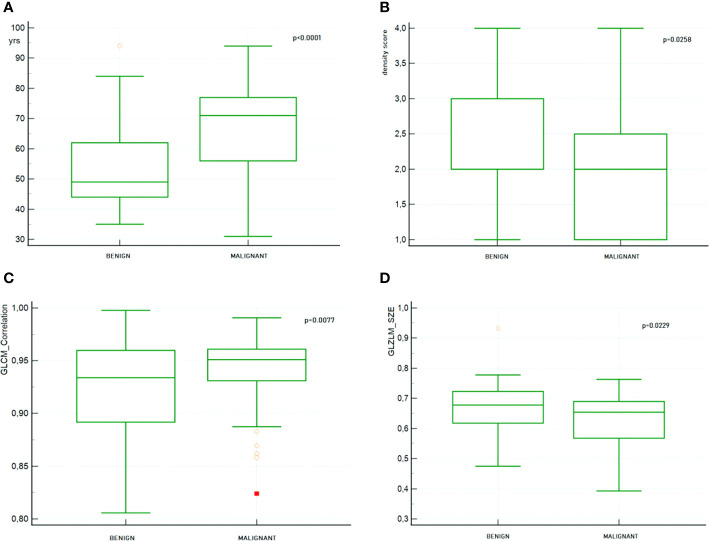
Boxplot together with the p value of 4 most representative selected features. **(A)** Patient’s age **(B)** Breast density **(C)** GLCM_Correlation **(D)** GLZLM_SZE.

In **Figure 8**, the distribution boxplot together with the p value of four most representative selected features is shown; two clinical and two radiomic, one of first and one of second order.

In **Supplementary Material** the decision tree for each predictive model (M1, M2 and M3) and the corresponding confusion matrices are reported.

Radiomic features provided by the three models showed significant differences (p < 0.05) between malignant and benign lesions.

**Figure 9:**
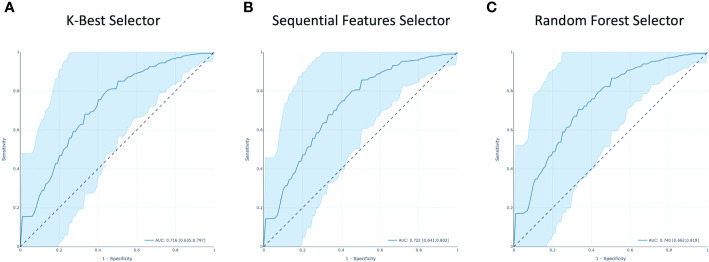
The receiver operating characteristic (ROC) curves of all the models. Area under the curve (AUC) is highlighted. **(A–C)** are respectively from KB, S and RF features selection methods.

In **Figure 9**, the ROC curves for model M1–3 are shown together with their AUC values. The M1 model yielded an AUC value of 0.716 with confidence interval (CI) of [0.635–0.797]. While an AUC value of 0.722 with CI [0.640–10.802] and 0.740 with CI [0.662–0.819] were found for the M2 and M3 models, respectively.

## 4 Discussion

In a recently published paper (20), it was shown that the proposed radiomic model could help reduce unnecessary biopsies. In fact, especially in the presence of architectural distortion, DBT can detect some apparently benign lesions as suspicious because of its high sensitivity.

Due to its widespread among hospitals and low economic impact, in Italy DBT is used as a first level screening and patients are eventually directed to MRI according to the radiologist’s opinion. So, even if the two methods can probably be complementary (9), DBT is by far the most common diagnostic method.

For this reason, we aimed to develop a model that could help radiologists in their first level diagnosis and eventually to address the patient to further exams.

Having a dataset with high dimensionality a process of feature selection is mandatory to avoid oversampling. In fact, high-dimensional datasets are not preferred because they have lengthy training time and have high risk of overfitting. Feature selection helps to mitigate these problems by selecting features that have high importance to the model so that the data dimensionality can be reduced without much loss of the total information.

In this study, three feature selection methods were used, and consequently, three predictive models were derived. The resulting diagnostic performances of the 3 models are quite similar. The model derived from the RF Selector showed a slightly better performance with respect to the KB and S Selectors yielding AUC values of 0.740 [CI 0.662–0.819], 0.716 [CI 0.635–0.797], and 0.722 [CI 0.641–0.802], respectively.

The best diagnostic performance of the derived models is in accordance with other studies (3, 9, 21) but lower than the one obtained by Niu 2022. This last study enrolled a higher number of patients (185), and besides the tumor itself, the peritumoral areas were found to have a high discriminative power and were subsequently analyzed. However, the study was conducted on a cohort of patients having a very homogenous breast density, a feature that probably impinged on robustness.

It is worth noting that due to the fact that patients enrolled in our study belong to a screening protocol, the mean age was 60.61 ± 15.51 years. This is probably the reason why patients with benign lesions, usually younger, appear to have a higher parenchymal density score. In fact, in our population a significant inverse correlation was found between age and density (p < 0.0001) indicating that age somehow is disguising the density effect.

Some limitations of this study need to be highlighted.

Even if we evaluated the reproducibility of the delineation, feature stability inside repeated contours has not been assessed.

Moreover, in the feature selection step the variance has not been taken into account. In fact, due to the characteristics of the studied population some relevant features would have been excluded, as it was retrospectively investigated.

In addition, we derived our models from a relatively small sample size that could be hopefully augmented. Also, the choice of selecting exams performed by the same mammographer is limiting, and more data from patients enrolled in screening protocols in our institute could be exploited thus overcoming differences in DBT scanner by performing data harmonization. A better model could be constructed using an external validation set. For these reasons, we are designing a wider clinical trial in which, besides including in the delineation also of the peritumoral area, more hospitals will be involved with the aim of building a model that can be shared and whose robustness can be proven among different users.

In conclusion, according to the results obtained in our study, we think that the derived models could be considered as an aid to the radiologist in the diagnosis of breast tumor, at least at a first level screening, due to the good performance shown by the constructed models.

## Data availability statement

The raw data supporting the conclusions of this article will be made available by the authors, without undue reservation.

## Ethics statement

The studies involving human participants were reviewed and approved by Comitato Etico Centrale IRCCS Lazio Sezione IFO. Written informed consent for participation was not required for this study in accordance with the national legislation and the institutional requirements.

## Author contributions

FM and VL: study design, FM and VL: study conduct, LG and FF: tumor delineation and radiological assessment, AR and LP: anatomo-pathological data support, PO database implementation, FM: data collection, FM and VL: data processing, FM and VL: statistical data analysis, FM and VL: drafting manuscript, AV and PO: manuscript revision. All authors contributed to the article and approved the submitted version.
