# Supplementary material for: Clinical-radiomic models based on digital breast tomosynthesis images: a preliminary investigation of a predictive tool for cancer diagnosis
Source: Front Oncol. 2023 May 12;13:1152158. doi: 10.3389/fonc.2023.1152158 (PMC10213670; doi:10.3389/fonc.2023.1152158)
Supplement: Supplementary file 1 [file DataSheet_1.docx]

Supplementary Material

**Clinical-Radiomic models based on digital breast tomosynthesis images: a preliminary investigation for a predictive tool for cancer diagnosis**

**Running title: DBT radiomic in cancer diagnosis**

Murtas F.^1,2^, Landoni V.^1*^, Ordonez P.^1^, Greco L.^3^, Ferranti F.R.^3^, Russo A.^4^, Perracchio L.^4^, Vidiri A.^3^

^1^ Medical Physics Dept., IRCCS Regina Elena National Cancer Institute, Rome, Italy.

^2^ Department of Biomedicine and Prevention, University of Rome "Tor Vergata", Rome, Italy.

^3^ Radiology and Diagnostic Imaging Dept., IRCCS Regina Elena National Cancer Institute, Rome, Italy.

^4^ Pathology Dept., IRCCS Regina Elena National Cancer Institute, Rome, Italy.

*** Correspondence:**Corresponding Author
valeria.landoni@ifo.it

Keywords: Radiomic_1_, predictive model_2_, breast cancer_3_, AI_4_, tomosynthesis (DBT)_5_.

## Supplementary Figures

**Supplementary Figure 1.** Example of the decision tree classifier obtained with RF based machine learning algorithm and K-Best based features selection process.

**Supplementary Figure 2.** Example of the decision tree classifier obtained with RF based machine learning algorithm and SFS based features selection process.

**Supplementary Figure 3.** Example of the decision tree classifier obtained with both feature selection and machine learning algorithm based on Random Forrest.

**
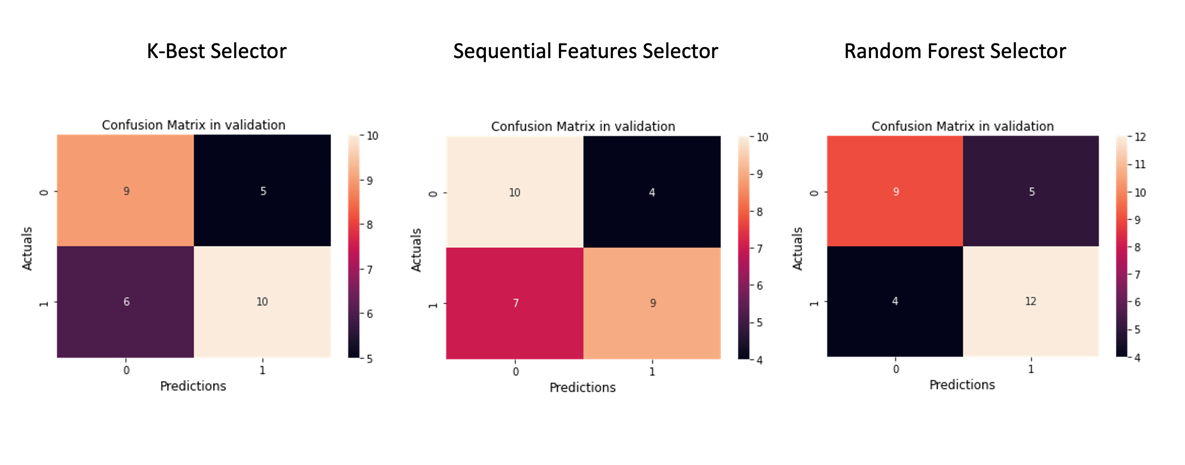
**

**Supplementary Figure 4.** Confusion matrices obtained using the validation set for Model 1, Model 2 and Model 3 respectively.
